# Increased Ectodomain Shedding of Cell Adhesion Molecule 1 from Pancreatic Islets in Type 2 Diabetic Pancreata: Correlation with Hemoglobin A1c Levels

**DOI:** 10.1371/journal.pone.0100988

**Published:** 2014-06-25

**Authors:** Takao Inoue, Man Hagiyama, Azusa Yoneshige, Takashi Kato, Eisuke Enoki, Osamu Maenishi, Takaaki Chikugo, Masatomo Kimura, Takao Satou, Akihiko Ito

**Affiliations:** Department of Pathology, Faculty of Medicine, Kinki University, Osaka, Japan; Okayama University, Japan

## Abstract

Pulmonary emphysema and type 2 diabetes mellitus (T2DM), both caused by lifestyle factors, frequently concur. Respectively, the diseases affect lung alveolar and pancreatic islet cells, which express cell adhesion molecule 1 (CADM1), an immunoglobulin superfamily member. Protease-mediated ectodomain shedding of full-length CADM1 produces C-terminal fragments (CTFs) with proapoptotic activity. In emphysematous lungs, the CADM1 shedding rate and thus the level of CTFs in alveolar cells increase. In this study, CADM1 expression in islet cells was examined by western blotting. Protein was extracted from formalin-fixed, paraffin-embedded sections of pancreata isolated from patients with T2DM (n = 12) or from patients without pancreatic disease (n = 8) at autopsy. After adjusting for the number of islet cells present in the adjacent section, we found that full-length CADM1 decreased in T2DM islets, while ectodomain shedding increased. Hemoglobin A1c levels, measured when patients were alive, correlated inversely with full-length CADM1 levels (*P* = 0.041) and positively with ectodomain shedding rates (*P* = 0.001). In immunofluorescence images of T2DM islet cells, CADM1 was detected in the cytoplasm, but not on the cell membrane. Consistently, when MIN6-m9 mouse beta cells were treated with phorbol ester and trypsin to induce shedding, CADM1 immunostaining was diffuse in the cytoplasm. When a form of CTFs was exogenously expressed in MIN6-m9 cells, it localized diffusely in the cytoplasm and increased the number of apoptotic cells. These results suggest that increased CADM1 ectodomain shedding contributes to blood glucose dysregulation in T2DM by decreasing full-length CADM1 and producing CTFs that accumulate in the cytoplasm and promote apoptosis of beta cells. Thus, this study has identified a molecular alteration shared by pulmonary emphysema and T2DM.

## Introduction

Type 2 diabetes mellitus (T2DM) is caused by peripheral resistance to insulin and an inadequate secretory response by pancreatic beta cells. As such, patients have “relative” insulin deficiency. Several pathophysiological conditions are believed to contribute to the beta cell dysfunction. One is glucotoxicity, in which the continuous overstimulation of beta cells by glucose eventually leads to the depletion of insulin stores, worsening of hyperglycemia, and deterioration of beta cell function [1]. A second is lipotoxicity, in which persistently elevated free fatty acids contribute to beta cell failure [1]. Yet another is alteration of the incretin system: the insulinotropic actions of glucose-dependent insulinotropic polypeptide are significantly diminished and the secretion of glucagon-like peptide 1 is deficient in T2DM patients [2]–[4]. Consistent with the nature of these pathophysiological conditions, many studies of beta cell failure in T2DM have focused on environmental abnormalities outside pancreatic islets. By contrast, molecular alterations within T2DM islet cells that may be responsible for disease development have not been fully elucidated. This may be due to the limited availability of fresh pancreatic tissues from T2DM patients.

T2DM is a common complex disease where multiple genetic and environmental factors have an intricate interplay. Recent researches based on genome-wide association studies and comparative gene expression assays have identified dozens of susceptibility loci and candidate genes responsible, such as *transcription factor 7-like-2*, *potassium voltage-gated channel, KQT-like subfamily, member 1* and *aryl hydrocarbon nuclear receptor translocator* [5]–[7]. Environmental factors involved in the pathogenesis include a sedentary lifestyle, and high-fat dietary and cigarette smoking habits [8], [9]. Cigarette smoking is also an independent risk factor for atherosclerotic cardiovascular [10] and chronic airway diseases, as typically exemplified by emphysema [11], a pulmonary disease that features alveolar wall destruction, resulting in enlarged airspaces and loss of surface area for gas exchange.

Cell adhesion molecule 1 (CADM1), also known as tumor suppressor in lung cancer 1 (TSLC1), is an intercellular adhesion molecule that belongs to the immunoglobulin superfamily [12]. The membrane-spanning glycoprotein is composed of three extracellular Ig-like domains, a single transmembrane region, and a short carboxy-terminal intracytoplasmic tail with a protein 4.1 interaction sequence and a PDZ type II domain-binding motif [12]. Various cell types express CADM1, including lung and biliary epithelial cells, nerve cells, mast cells [13]–[15], and pancreatic islet endocrine cells [16]. In mice, CADM1 expression is restricted to alpha cells, whereas it is expressed equally in the four cell types (alpha, beta, D, and PP) that constitute human islet cells [16]. CADM1 appears to play a significant role in hormone secretion by mediating islet cell-cell and islet cell-nerve interactions through *trans*-homophilic binding [16], [17]. Recent studies have shown that CADM1 expression is regulated by post-transcriptional mechanisms, including glycosylation and proteolytic cleavage, referred to as shedding [18], [19]. CADM1 is cleaved at one of two sites in its ectodomain, yielding two membrane-associated C-terminal fragments, αCTF and βCTF. CADM1 ectodomain shedding appears to occur on the plasma membrane: shedding proceeds in isolated plasma membranes, and it is directly mediated by a membrane-bound metalloprotease called a disintegrin and metalloproteinase 10 [19]. We recently identified CADM1 shedding as a key event in the development of pulmonary emphysema [20]. CADM1 ectodomain shedding rates increase in emphysematous lungs, and the mitochondrial localization of αCTF contributes to lung epithelial cell apoptosis [20].

In 2011, Rodríguez-Rigueiro *et al.* described a novel procedure for protein extraction from formalin-fixed, paraffin-embedded tissues [21]. The technique is pivotal because protein expression in archived pathological specimens from autopsied patients can now be assessed in a quantitative manner. In this study, we extracted proteins from paraffin sections of pancreata that were removed at autopsy from patients with T2DM or patients without pancreatic disease. Western blot analysis and histological examination showed altered expression of CADM1 in T2DM islet cells. We also analyzed the association between alterations in CADM1 expression and hemoglobin (Hb) A1c levels. Our results identify increased CADM1 ectodomain shedding as a molecular event shared by pulmonary emphysema and T2DM.

## Materials and Methods

### Ethics statement

The Ethics Committee of Kinki University approved the experimental protocol and waived the need for written informed consent (25–088).

### Antibodies and reagents

A rabbit anti-CADM1 polyclonal antibody directed against the C-terminal 15-amino acid peptide was generated in our laboratory and described previously [22]. Other primary antibodies targeted glucagon (mouse monoclonal; Sigma-Aldrich, St. Louis, MO, USA), insulin (guinea pig polyclonal; Dako, Glostrup, Denmark), and β-actin (mouse monoclonal AC-15; Sigma-Aldrich). Peroxidase- and fluorophore-conjugated secondary antibodies were obtained from Amersham (Buckinghamshire, England) and Jackson ImmunoResearch (West Grove, PA, USA), respectively. Phorbol 12-myristate 13-acetate (PMA) and trypsin were purchased from Wako Pure Chemical Industries (Osaka, Japan).

### Cell culture and transfection

MIN6-m9 mouse islet beta cells, kindly provided by Professor S. Seino of Kobe University, Japan [23], were grown in Dulbecco's modified Eagle medium (DMEM, 4.5 g/L glucose; Wako) supplemented with 10% fetal bovine serum, 100 units/mL penicillin, 100 µg/mL streptomycin, 5 mM HEPES buffer, and 50 µM 2-mercaptoethanol at 37°C in 5% CO_2_/95% air. To induce CADM1 ectodomain shedding, MIN6-m9 cells were incubated in DMEM containing 200 nM PMA and 0.025% w/v trypsin (a concentration too low to induce cell detachment) for 45 min, as described previously [20]. The pCX4bsr plasmid vector expressing CADM1-αCTF, named pCX4bsr-SP-αCTF, was constructed previously [20]. We also constructed the plasmid vector expressing a mutant form of αCTF, αCTFmut, carrying 11 amino acid substitutions and 2 amino acid deletions in the intracytoplasmic domain of αCTF (pCX4bsr-SP-αCTFmut) [20]. In lung epithelial cells, αCTF localized to mitochondria and induced apoptosis, whereas αCTFmut did neither [20]. For exogenous expression of αCTF or αCTFmut, MIN6-m9 cells were grown to 60%–70% confluence and transfected with pCX4bsr-SP-αCTF or pCX4bsr-SP-αCTFmut using Lipofectamine LTX and PLUS reagents (Invitrogen, Carlsbad, CA, USA) according to the manufacturer’s instructions.

### Human samples

We reviewed clinical records, autopsy records, and pathological specimens archived in Kinki University Hospital (Osaka, Japan) to identify autopsied patients who had been diagnosed with T2DM and who had no clinical or histological indications of insulitis, acute or chronic pancreatitis, or pancreatic neoplasm. Twelve patients who met this criterion were autopsied in the last six years (approximately two patients per year) (T2DM group). For controls, we selected eight patients who died in the last six years (approximately one to two patients per year) of diseases without clinical or histological evidence of pancreatic involvement (control group). All patients were autopsied within a couple of hours after death. Pancreata removed at autopsy were fixed with 10% buffered formalin for several days. A cross-sectional segment of the middle portion was then excised, embedded in paraffin, and cut serially into 20-µm- and 3-µm-thick sections for protein extraction and histological examination, respectively. HbA1c levels were measured when the patients received outpatient or inpatient care at Kinki University Hospital. The Ethics Committee of Kinki University approved the experimental protocol (25–088).

### Protein extraction and western blot analysis

Protein was extracted from a 20-µm-thick section of paraffin-embedded pancreas according to the method described by Rodríguez-Rigueiro *et al*. [21] with minor modifications. Briefly, the section was deparaffinized by incubation in mineral oil at 95°C for 2 min, and the supernatant was removed by centrifugation at 11320×*g*. The pellet was washed serially with phosphate-buffered saline and citrate-SDS buffer containing 200 mM Tris-HCl (pH 7.5), 200 mM NaCl, 5% SDS, and 100 mM sodium citrate. Protein was extracted from the pellet by incubation at 100°C for 20 min and at 80°C for 2 h in citrate-SDS buffer with occasional vortexing and was collected in the supernatant after centrifugation at 11320×*g* for 15 min at room temperature. Protein was extracted from cultured cells as described previously [16]. After sample preparation and western blot analysis, immunoreactive band intensities were quantified using ImageJ (National Institutes of Health, Bethesda, MD, USA), as described previously [24].

### Immunofluorescence, islet cell count, and mitochondrial labeling

Immunofluorescence double staining of pancreas sections (3-µm thick) adjacent to those used for protein extraction was performed as described previously [16], using primary antibodies against CADM1, insulin, and glucagon. Tissue areas were measured using the ACT2U software attached to an Eclipse E1000M microscope (Nikon, Tokyo, Japan). The pancreatic tissue area in individual sections ranged from 1.3 to 3.6 cm^2^. The tissue area was scanned with a confocal laser microscope (AZ-C2+; Nikon), and the number of alpha cells (glucagon-positive), beta cells (insulin-positive), and total islet cells (identified by DAPI nuclear staining), as well as the number of alpha and beta cells with cell-membranous staining for CADM1, was counted manually. Small islets (< approximately 50 µm in diameter) were excluded because they were often difficult to recognize due to few positive cells. For each section, the islet cell count (/cm^2^) was calculated as the cell number divided by the tissue area, and the proportion of cells with CADM1 cell-membranous staining (%) was calculated by dividing the number of stained cells by the total cell number. The mean and standard error (SE) of the values were calculated for the control (n = 8) and T2DM (n = 12) groups. Immunofluorescence coupled with mitochondrial labeling of MIN6-m9 cells was performed as described previously [20], using the anti-CADM1 antibody and MitoTracker (Molecular Probes, Eugene, OR, USA).

### Insulin secretion assay

Induction of insulin secretion was performed according to the published protocol [25], [26]. MIN6-m9 cells were transfected in 24-well plates 2 days prior to the assay. After washed with Krebs-Ringer-bicarbonate (KRB; 133 mM NaCl, 4.7 mM KCl, 5 mM NaHCO_3_, 2.5 mM CaCl_2_, 1.2 mM MgSO_2_, 1.2 mM KH_2_PO_4_, 10 mM HEPES, and 0.5% bovine serum albumin) buffer, cells were incubated in 500 µl of KRB buffer containing 2.0 mM glucose at 37°C for 30 min, and then 100 µl of the media were collected (t = 0). The same volume of KRB buffer containing 117 mM glucose (final concentration, 25 mM) was added to each well, incubated at 37°C, and then 30 µl of the media were collected at three timepoints (t = 15 min, 1 h and 2 h). The collected media were centrifuged at 11320×*g* for 2 min, and insulin concentrations in the supernatants were measured with a mouse insulin ELISA kit (S-type, Shibayagi Co., Ltd., Shibukawa, Japan) according to the manufacturer’s instructions. Immediately after the medium collection at t = 2 h, cells were harvested by trypsin and EDTA treatment, washed with phosphate buffer, and lysed in a buffer containing 50 mM Tris-HCl (pH 8.0), 150 mM NaCl, 1% TritonX-100, and protease inhibitor cocktail (Sigma). Protein concentrations in the lysates were measured with BCA™ Protein Assay Kit (Thermo Scientific, Waltham, MA, USA). Glucose-induced insulin secretion capacity was expressed as a ratio (fold increase) relative to the baseline value [(t = 15 min, 1 h, or 2 h)/(t = 0)] and normalized to the protein concentration (i.e., cell volume). The mean and SE of the ratio were calculated from triplicate experiments for each cell type. Assays were repeated three times, with essentially similar results.

### Terminal deoxynucleotidyl transferase-mediated dUTP nick end labelling (TUNEL)

TUNEL assays were conducted on MIN6-m9 cells using the In Situ Cell Death Detection Kit (Roche Applied Science, Upper Bavarie, Germany) according to the manufacturer’s instructions, as we described previously [20]. Briefly, after 2 days of transfection, the number of TUNEL-positive cells was counted in more than 500 cells identified by nuclear counterstaining with DAPI. The mean and SE of the proportion of TUNEL-positive cells were calculated from triplicate experiments for each cell type. Assays were repeated three times, with essentially similar results.

### Statistical analysis

Clinical and histological variables in the control and T2DM groups were assessed with the Mann-Whitney *U-*test for continuous variables and the Fisher’s exact test for categorical variables. Statistical differences between the two groups were analyzed using the Mann-Whitney *U*-test to assess quantified western blot data and Student’s *t*-test to assess islet cell counts and proportions, insulin secretion capacity, and TUNEL-positive cell proportions. Correlations were analyzed with Spearman’s rank test. A *P*-value ≤0.05 was considered significant.

## Results

### Increased CADM1 ectodomain shedding in diabetic pancreata

**Figure 1:**
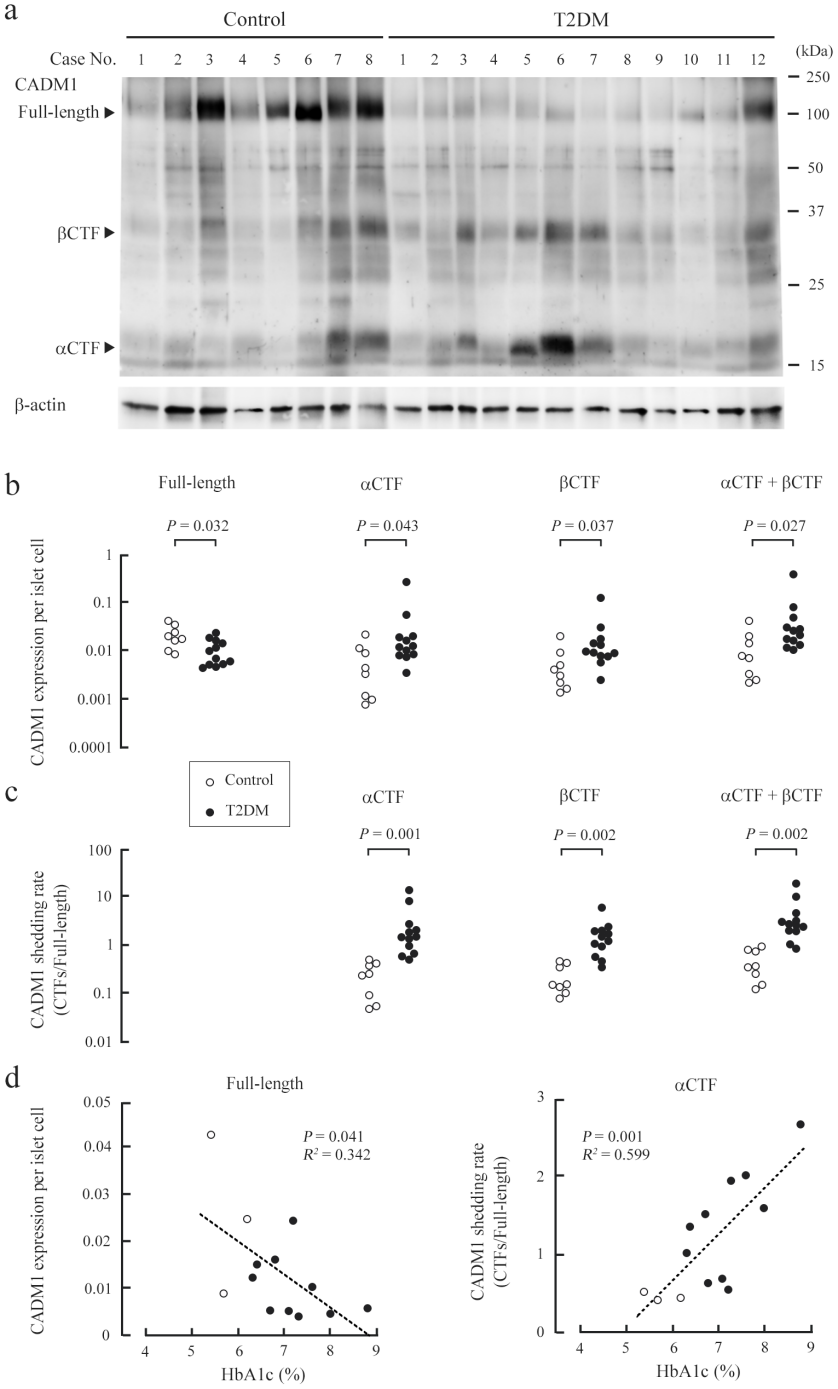
Increased ectodomain shedding of CADM1 in T2DM pancreata. (**a**) Western blot analysis of CADM1 expression in control and T2DM pancreata. Cases are numbered as in Table S1. Arrowheads indicate bands corresponding to the full-length, αCTF, and βCTF forms of CADM1. The blot was reprobed with an anti-β-actin antibody to show protein loading. (**b**) CADM1 expression per islet cell in each patient sample. For each lane in **a**, the intensities of the full-length CADM1, αCTF, βCTF, and β-actin bands were quantified, and CADM1 levels were expressed relative to β-actin and the islet cell count (/cm^2^ of tissue). Statistical significance was analyzed with the Mann-Whitney *U*-test. *P*-values are shown. (**c**) CADM1 ectodomain shedding rates (relative amounts of CTFs to the full-length CADM1). Statistical significance was analyzed with the Mann-Whitney *U*-test. *P*-values are shown. (**d**) Correlation of HbA1c levels with full-length CADM1 expression per islet cell (left) or the CADM1 shedding rate (αCTF/full-length; right). In each graph, the dot distribution approximated a linear function (dotted lines). Correlations and statistical significance were analyzed with Spearman’s rank test. *R^2^* and *P*-values are shown.

As described in the previous section, we selected 12 autopsied patients with T2DM (T2DM group) and eight autopsied control patients (control group) for this study (Table 1 and Table S1). There was no difference in age or sex between the two groups. The number of beta and total islet cells per cm^2^ of tissue in T2DM pancreata was approximately half that in control pancreata (Table 1), consistent with previous findings [27]. The number of alpha cells was also decreased in T2DM pancreata (Table 1). This finding is consistent with some past reports [28], [29], but inconsistent with or contrary to others [27], [30], [31], probably because of sampling bias in our study where small islets (< approximately 50 µm in diameter) were excluded [29]. We extracted protein from pancreas sections, and analyzed the extracts by western blotting using a CADM1 antibody and a β-actin antibody to verify successful protein extraction (Fig. 1a). Consistent with our previous report [20], the CADM1 antibody detected three forms of CADM1 at approximately 100, 35, and 18 kDa, corresponding to full-length CADM1, βCTF, and αCTF, respectively (Fig. 1a). We previously reported that CADM1 distributed mainly to islet cells and also to nerves in the human pancreas [16]. Thus, islet cells were considered the major cell source of CADM1. CADM1 expression per islet cell was estimated by normalizing the band intensity of each CADM1 form to β-actin and dividing by the number of islet cells per cm^2^ of tissue, determined by analyzing the adjacent section (Table S1). Full-length CADM1 expression per islet cell was lower in the T2DM group than in the control group, whereas βCTF and αCTF expression per islet cell was higher in the T2DM group (Fig. 1b). The statistical significance of the differences in expression was marginal. However, the CADM1 shedding rates, calculated as αCTF/full-length CADM1, βCTF/full-length CADM1, and (αCTF + βCTF)/full-length CADM1, were higher in the T2DM group, and the differences were statistically significant (Fig. 1c). Full-length CADM1 expression inversely correlated with the three shedding rates (Figure S1). These results suggest that increased ectodomain shedding contributes to the decreased levels of full-length CADM1 in T2DM pancreata.

**Table 1:** Characteristics of the patient groups^a^.

Variables	Control (n = 8)	T2DM (n = 12)	*P*-value
Age (range, median)	48–86, 69.9	52–82, 69.4	0.729
Sex (M/F)	6/2	8/4	1
Islet cell count (/cm^2^)^b^			
alpha	3576±681.5	1800±389.9	0.017
beta	5401±965.5	2246±398.1	0.014
total	15069±2983.0	7571±1126.1	0.043
% Islet cells with CADM1 cell-membranous staining^b^			
alpha	49.5±2.19	13.6±3.53	<0.001
beta	81.3±1.24	22.3±6.16	<0.001
HbA1c (%)^b^^,^ c	5.8±0.23	7.3±0.25	0.007
(mmol/mol)	(39.7±2.33)	(56.4±2.75)	

a Data for individual patients are presented in Table S1.

b Data are expressed as the mean ± SE.

c Control, n = 3; T2DM, n = 10.

### Correlation between the CADM1 shedding rate and HbA1c levels

We collected HbA1c data from the clinical records of the autopsied patients. Data were available for all but two patients in the T2DM group. For each T2DM patient, the mean was calculated from measurements taken in the six months before death, with measurements taken two weeks before death omitted because they might have been influenced by agonal phase treatment (Table S1). In the control group, HbA1c data for three patients was available, and the mean was calculated from measurements taken in the two years before death, excluding the last two weeks. The mean HbA1c levels inversely correlated with full-length CADM1 expression (*P* = 0.041) and positively correlated with CADM1 ectodomain shedding rates (*P* = 0.001 for αCTF/full-length CADM1 and *P* = 0.039 for (αCTF + βCTF)/full-length CADM1) (Fig. 1 and Figure S2).

### Subcellular localization of CADM1 in islet cells

**Figure 2:**
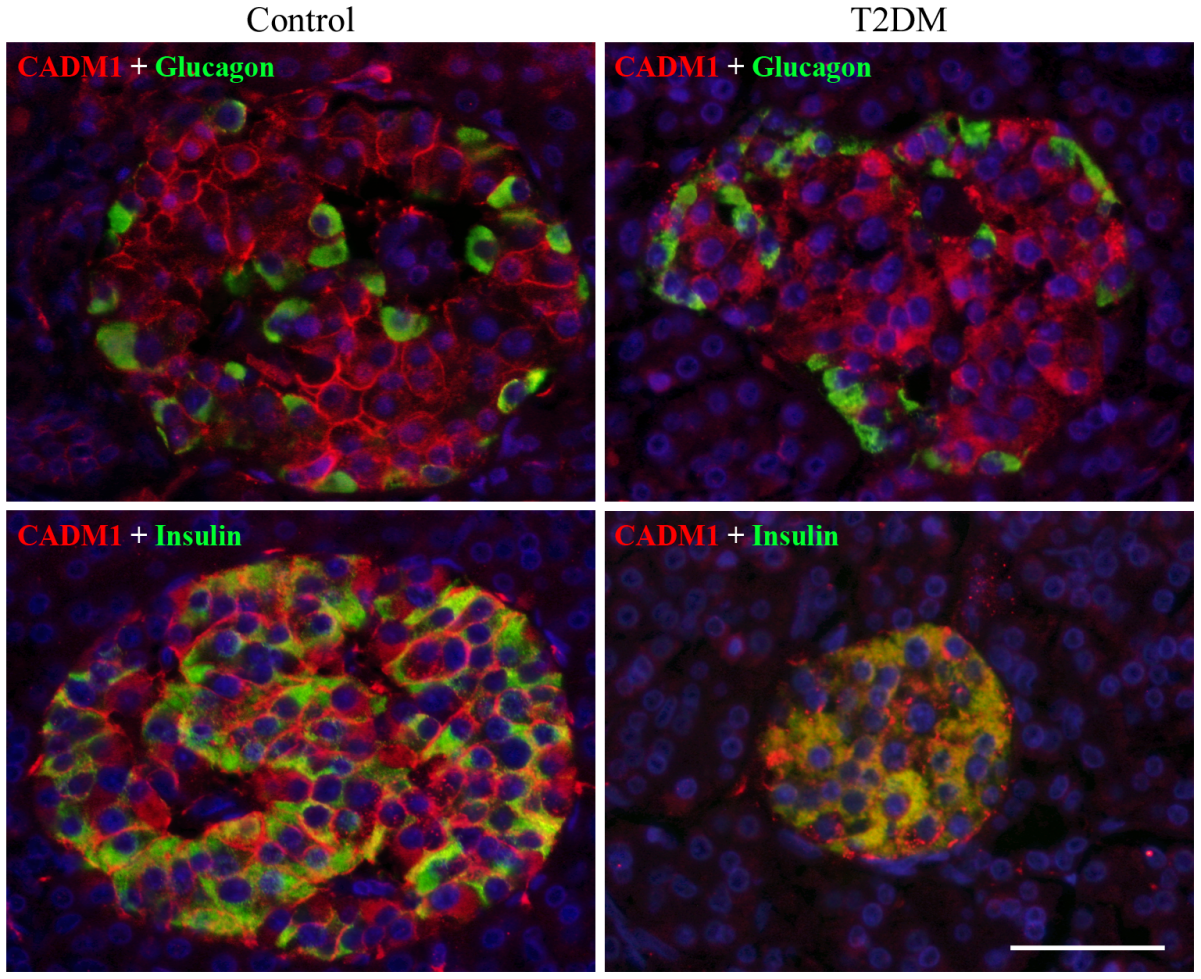
Representative photomicrographs of CADM1 immunofluorescence in pancreatic islets. Pancreatic sections from control (case 7; left) and T2DM (case 3; right) patients were double stained with antibodies against CADM1 (red) and glucagon (green; top) or insulin (green; bottom) and then counterstained with DAPI (blue). Merged images are shown. Bar  = 50 µm.

Pancreas sections were double stained with antibodies against CADM1 and glucagon or insulin. Consistent with our previous study [16], CADM1 staining was detected primarily on the cell membrane of most islet cells, including alpha (glucagon-positive) and beta (insulin-positive) cells, in control pancreata (Fig. 2). By contrast, in T2DM pancreata, diffuse cytoplasmic staining of CADM1, rather than cell-membranous staining, was prominent (Fig. 2). The proportion of alpha or beta cells with CADM1 cell-membranous staining in T2DM pancreata was only a quarter of that in control pancreata (Table 1).

**Figure 3:**
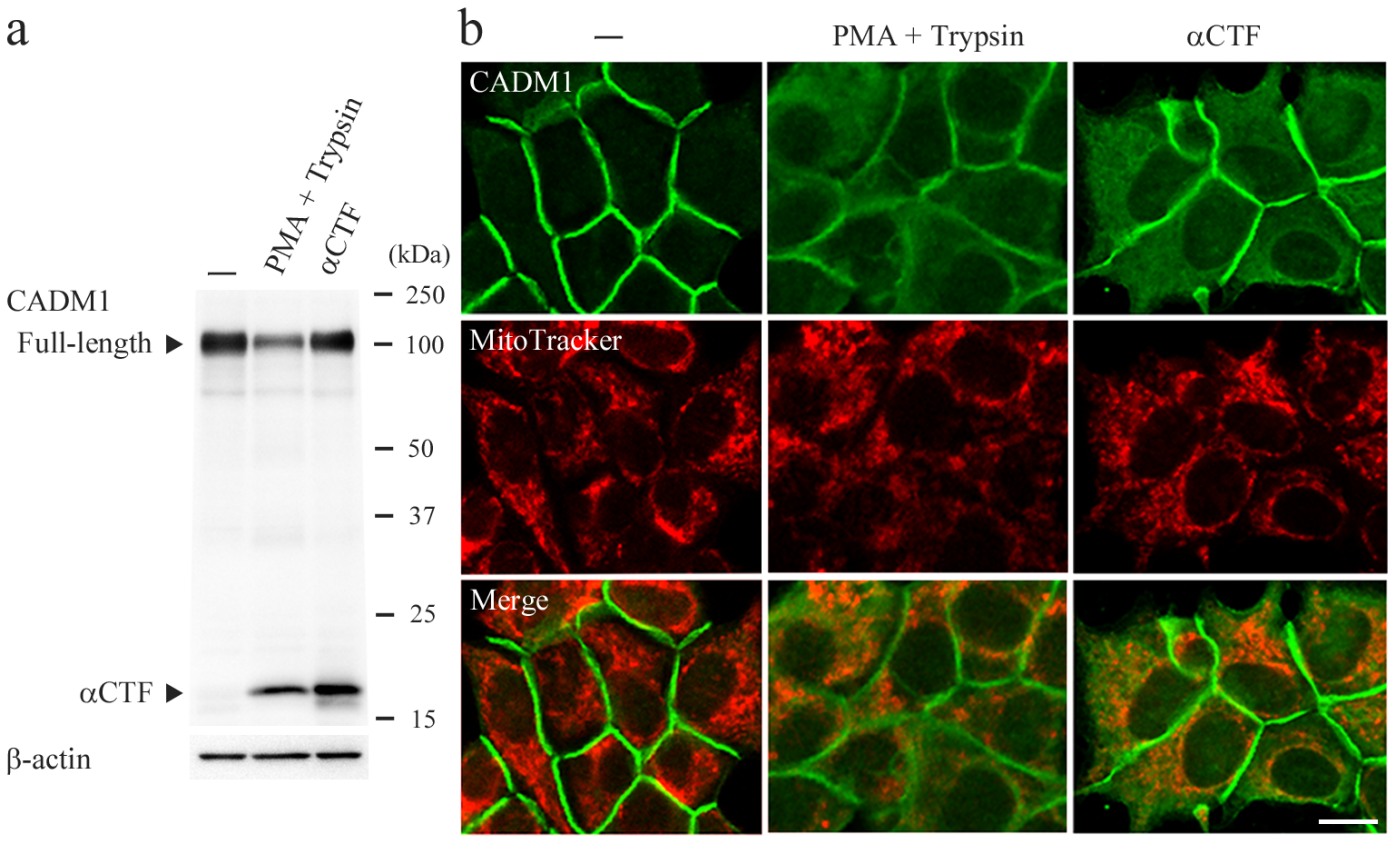
Subcellular localization of endogenous and exogenous αCTF in MIN6-m9 cells. MIN6-m9 cells were untreated (−), treated with PMA (200 nM) and trypsin (0.025% w/v), or transfected with pCX4bsr-SP-αCTF. After 45 min of treatment and 2 days of transfection, CADM1 levels were assessed in western blot (**a**) and immunofluorescence (**b**) analyses using a CADM1 antibody. In **a**, arrowheads indicate full-length CADM and αCTF. The blot was reprobed with an anti-β-actin antibody to show the protein loading. In **b**, cells were double stained with CADM1 antibody (green; top) and MitoTracker (red; middle). Merged images are also shown (bottom). Data are representative of three independent experiments. Bar  = 10 µm.

We recently showed that αCTF localized to mitochondria in lung epithelial cells [20]. Thus, CADM1 cytoplasmic staining in islet cells might derive from αCTF. To examine this possibility, we used MIN6-m9 mouse islet beta cells that expressed full-length CADM1 but not αCTF or βCTF under basal conditions (Fig. 3a). When treated with PMA and trypsin, the cells expressed αCTF, and the shedding rate was comparable to that observed in T2DM pancreata (Fig. 3a and Figure S3). In immunofluorescence analysis, CADM1 localized exclusively to the cell membrane in untreated cells (Fig. 3b). By contrast, in the treated cells, CADM1 cell-membranous staining decreased and diffuse cytoplasmic staining appeared (Fig. 3b). Similar cytoplasmic staining was observed in MIN6-m9 cells transfected with cDNA encoding αCTF (Fig. 3, a and b). Double-staining analysis did not reveal a significant amount of colocalization between CADM1 and MitoTracker signals in treated or transfected cells (Fig. 3b). These results suggest that αCTF is present throughout the cytoplasm of islet beta cells.

### Functional analyses of αCTF using MIN6-m9 cells

**Figure 4:**
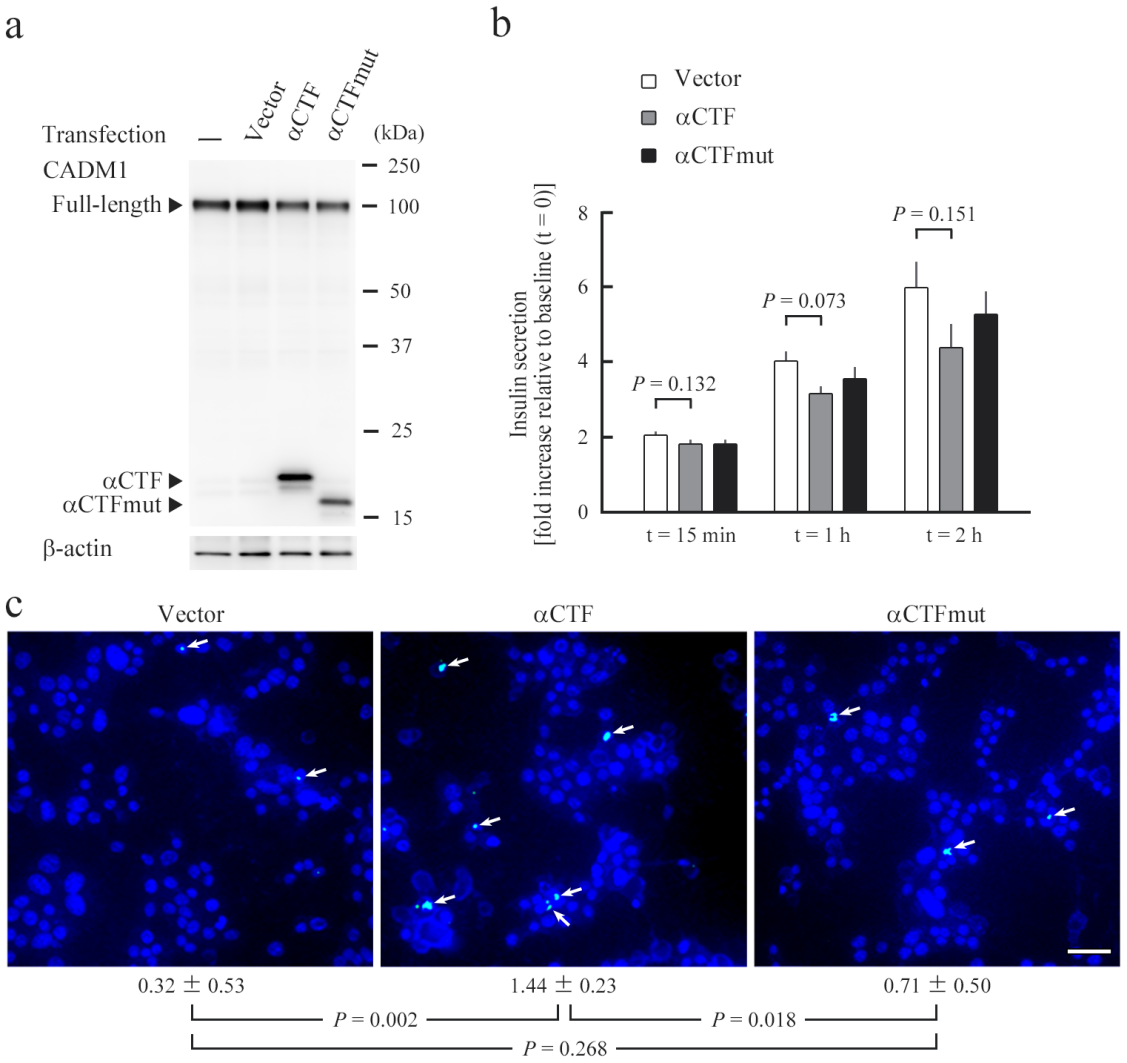
Functional analyses of αCTF in MIN6-m9 cells. MIN6-m9 cells were untreated (−), or transfected with the empty pCX4bsr vector, or the vector expressing either αCTF or αCTFmut. After 2 days of transfection, CADM1 levels were assessed in a western blot using a CADM1 antibody (**a**). Arrowheads indicate full-length CADM, αCTF, and αCTFmut. The blot was reprobed with an anti-β-actin antibody to show the protein loading. (**b**) Glucose-induced insulin secretion assay. The mean ratio (fold increase) relative to the baseline (t = 0) and normalized to the cell volume is plotted with a bar indicating SE. There was no difference between any pair at each timepoint; the smallest *P*-values by *t*-test are shown. (**c**) TUNEL assay. TUNEL and DAPI fluorescent stains are colored green and blue, respectively. TUNEL-positive cells are recognized by bluish white nuclei where the two stains merge (indicated by arrows). Mean ± SE of the percentage of TUNEL-positive cells and *P*-values by *t*-test are shown below the photomicrographs. Data in **b** and **c** are representative of three independent assays, respectively. Bar  = 50 µm.

MIN6-m9 cells were transfected with the plasmid vector encoding either αCTF or αCTFmut, a non-functional mutant form of αCTF [20], or the empty vector. Exogenous expression of αCTF and αCTFmut was confirmed by western blot analyses (Fig. 4a) and the double-staining analyses, showing αCTFmut to be present diffusely in the cytoplasm, similar to αCTF (Figure S4). After 2 days of transfection, cells were subjected to glucose-induced insulin secretion assays. No significant difference in the insulin secretion capacity was found among the three types of transfectants at any timepoint examined (15 min, 1 h, and 2 h after the shift from low to high glucose) (Fig. 4b). Another set of transfectants was examined for their frequency of apoptosis by TUNEL assays. Apoptosis occurred infrequently in these transfectants, but the percentage of TUNEL-positive cells was significantly higher in αCTF transfectants than in αCTFmut or vector transfectants (Fig. 4c).

## Discussion

In this study, we found that the level of full-length CADM1 in islet cells was lower in T2DM pancreata than in control pancreata. The decrease was associated with increased ectodomain shedding, suggesting that increased ectodomain shedding was a cause of decreased full-length CADM1. It remains unknown whether increased CADM1 ectodomain shedding in T2DM pancreata is a cause or result of the disease. In considering this question, it is noteworthy that *de novo* synthesis of diacylglycerol from glucose increases in hyperglycemic and diabetic environments, and subsequently protein kinase C (PKC) isozymes, such as PKCα, PKCβ1 and PKCβ2, are activated in various types of cells including endothelial cells [32]–[34]. This PKC activation appears to participate in the development of diabetic microvascular complications, because these complications could be attenuated by inhibitors of PKCβ isozymes [33], [34]. In pancreatic beta cells, PKCα is shown to become active and translocate to the cell membrane in response to a glucose stimulus [35]–[37]. In addition, similarly to CADM1, N-cadherin is shed at its ectodomain by ADAM10 in glioblastoma cells, and this process requires PKCα activity [38]. These lines of evidence suggest that CADM1 ectodomain shedding may be in an elevated state via PKC activation due to chronic hyperglycemia in T2DM.

Full-length CADM1 localizes primarily to the cell membrane [16], [39]. In T2DM pancreata, the decrease in full-length CADM1 was associated with its absence from the cell membrane in both alpha and beta cells (Table 1). Previously, we reported that CADM1 contributes to gap junctional communication among mouse islet alpha cells and that decreased expression of CADM1 on the cell membrane of mouse alpha cells resulted in the supernormal secretion of glucagon, suggesting that mouse CADM1 may serve as a volume limiter of glucagon secretion by sustaining alpha cell-cell attachment necessary for efficient gap junctional communication [17]. Similarly in human T2DM pancreata, downregulation of full-length CADM1 may impair gap junctional communication among alpha cells and thus underlie the hyperglucagonemia often observed in T2DM patients [40]. Gap junctional communication among beta cells and between alpha and beta cells may also be impaired in T2DM pancreata. This may lead to secretory malfunction in beta cells because gap junctional communication is important for the pulsatile and synchronous release of insulin from beta cells [41], [42]. In fact, the decrease in full-length CADM1 and the αCTF/full-length CADM1 rate were correlated with HbA1c levels across individual patients, suggesting a close relationship between blood glucose dysregulation and CADM1 shedding in T2DM. When exogenously expressed in MIN6-m9 cells, however, αCTF did not cause alterations in glucose-induced insulin secretion. Recent studies from several independent groups revealed that miR-375, an islet-specific microRNA, was overexpressed in T2DM pancreata [43]–[45]. This overexpression is postulated to participate in the pathogenesis of T2DM by downregulating its target genes including *Cadm1* [46]. Increased CADM1 ectodomain shedding may be important as another mechanism to downregulate full-length CADM1.

We recently reported that αCTF localizes to mitochondria and depolarizes the mitochondrial membrane potential in lung epithelial cells, leading to the induction of apoptosis [20]. In contrast, CADM1 ectodomain shedding products were present throughout the cytoplasm in T2DM islet cells. Consistently, αCTF showed a diffuse cytoplasmic localization in MIN6-m9 cells. Unexpectedly, we found that αCTF promoted apoptosis in MIN6-m9 cells. Although the frequency of apoptosis was low (1.44%), αCTF-mediated apoptotic pathway may have *in vivo* relevance in beta cell apoptosis that occurs with increased frequency in T2DM pancreata, because the number of beta cells lost by apoptosis is estimated to be at most 1 for every 3,750 beta cells per day [47], [48]. We are now examining where αCTF localizes in beta cells and how αCTF promotes beta cell apoptosis.

In conclusion, this study suggests that a local increase in protease activity on the cell membrane of pancreatic islet cells contributes to the pathogenesis of T2DM. Increased CADM1 ectodomain shedding is a possible pathogenic mechanism shared by pulmonary emphysema and T2DM, two diseases caused by unhealthy lifestyle factors, such as smoking and poor diet [49]. Interestingly, more than 10% of patients with T2DM also suffer from emphysema [50], [51]. There is a high incidence of concurrence because the pathophysiological conditions of one disease promote the other disease [52], [53]. The two diseases may also share a common pathogenic mechanism. Further investigation of CADM1 shedding in different cell types and diseases will provide deeper insight into key molecular mechanisms common to lifestyle-related diseases.

## Supporting Information

Figure S1 Scatter plots with dots indicating full-length CADM1 expression levels per islet cell and CADM1 shedding rates on the X and Y axes, respectively. The dot distribution was well approximated as linear (dotted lines) in each plot. Correlation and statistical significance were analysed by the Spearman’s rank test, and *R^2^* and *P*-values are shown.
(PDF)

Figure S2 Scatter plots with dots indicating HbA1c levels and CADM1 shedding rates (βCTF/full-length or αCTF + βCTF/full-length) on the X and Y axes, respectively. The dot distribution was well approximated as linear (dotted lines) in each plot. Correlations and statistical significance were analysed by the Spearman’s rank test, and *R^2^* and *P*-values are shown.
(PDF)

Figure S3 Graphs plotted with dots indicating CADM1 ectodomain shedding rates (αCTF/full-length). The data of control and T2DM pancreata are identical to Fig. 1c of the main text. MIN6-m9 cells were treated with PMA and trypsin, and then were subjected to Western blot and immunofluorescence analyses (see Fig. 3 of the main text). Experiments were repeated independently three times; the data from the three Western analyses are plotted (triangle). Statistical significance was analysed by the Mann–Whitney *U*-test, and *P*-values are shown.
(PDF)

Figure S4 Subcellular localization of αCTFmut, a mutant form of αCTF, in MIN6-m9 cells. MIN6-m9 cells were transfected with either the empty pCX4bsr vector or pCX4bsr-SP-αCTFmut. After 2 days of transfection, cells were double stained with a CADM1 antibody (green; top) and MitoTracker (red; middle). Merged images are also shown (bottom). Data are representative of three independent experiments. Bar  = 10 µm.
(PDF)

Table S1 **Characteristics of autopsied patients.**
(DOCX)
