# Supplementary figures and images for: Increased Ectodomain Shedding of Cell Adhesion Molecule 1 from Pancreatic Islets in Type 2 Diabetic Pancreata: Correlation with Hemoglobin A1c Levels
Source: PLoS One. 2014 Jun 25;9(6):e100988. doi: 10.1371/journal.pone.0100988 (PMC4071031; doi:10.1371/journal.pone.0100988)

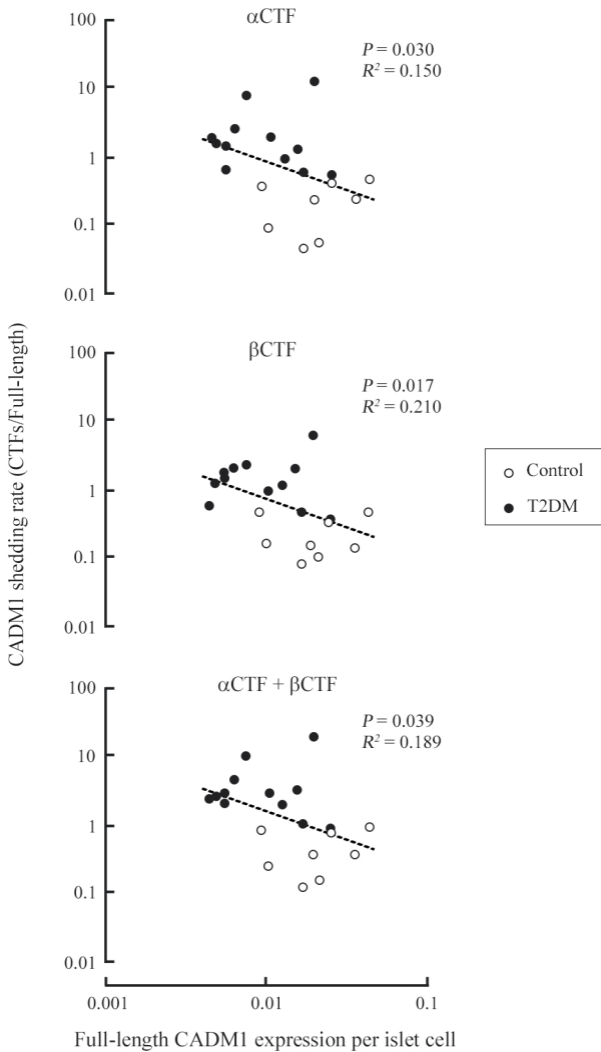

Supplement: Figure S1 — Scatter plots with dots indicating full-length CADM1 expression levels per islet cell and CADM1 shedding rates on the X and Y axes, respectively. The dot distribution was well approximated as linear (dotted lines) in each plot. Correlation and statistical significance were analysed by the Spearman’s rank test, and R2 and P-values are shown. (PDF) [file pone.0100988.s001.pdf]

CADM1 shedding rate (CTFs/Full-length)

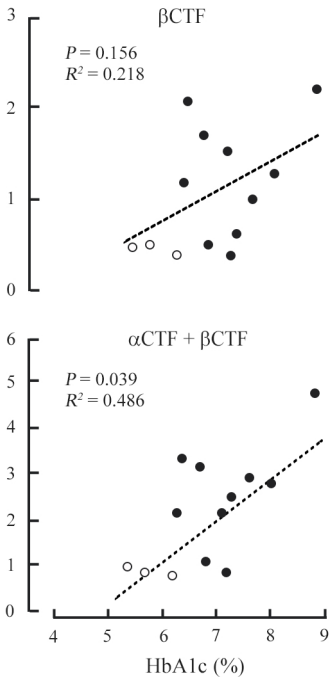

Supplement: Figure S2 — Scatter plots with dots indicating HbA1c levels and CADM1 shedding rates (βCTF/full-length or αCTF + βCTF/full-length) on the X and Y axes, respectively. The dot distribution was well approximated as linear (dotted lines) in each plot. Correlations and statistical significance were analysed by the Spearman’s rank test, and R2 and P-values are shown. (PDF) [file pone.0100988.s002.pdf]

CADM1 shedding rate  
(CTFs/Full-length)

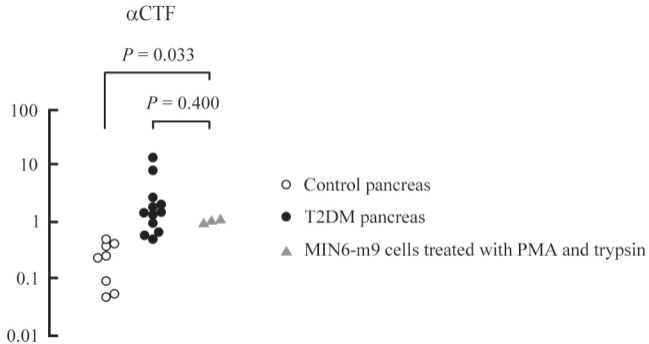

Supplement: Figure S3 — Graphs plotted with dots indicating CADM1 ectodomain shedding rates (αCTF/full-length). The data of control and T2DM pancreata are identical to Fig. 1c of the main text. MIN6-m9 cells were treated with PMA and trypsin, and then were subjected to Western blot and immunofluorescence analyses (see Fig. 3 of the main text). Experiments were repeated independently three times; the data from the three Western analyses are plotted (triangle). Statistical significance was analysed by the Mann–Whitney U-test, and P-values are shown. (PDF) [file pone.0100988.s003.pdf]

Vector

$\alpha$ CTFmut

CADM1

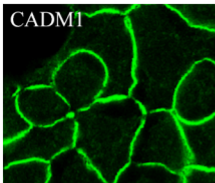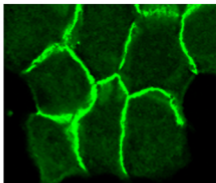

MitoTracker

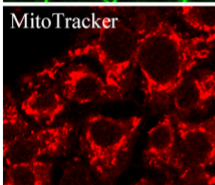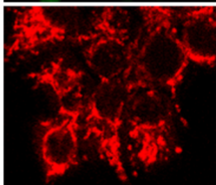

Merge

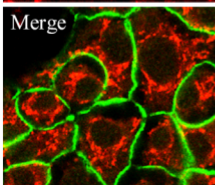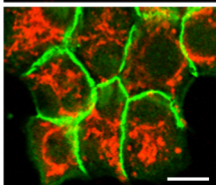

Supplement: Figure S4 — Subcellular localization of αCTFmut, a mutant form of αCTF, in MIN6-m9 cells. MIN6-m9 cells were transfected with either the empty pCX4bsr vector or pCX4bsr-SP-αCTFmut. After 2 days of transfection, cells were double stained with a CADM1 antibody (green; top) and MitoTracker (red; middle). Merged images are also shown (bottom). Data are representative of three independent experiments. Bar = 10 µm. (PDF) [file pone.0100988.s004.pdf]
