# Supplementary material for: Increased Ectodomain Shedding of Cell Adhesion Molecule 1 from Pancreatic Islets in Type 2 Diabetic Pancreata: Correlation with Hemoglobin A1c Levels
Source: PLoS One. 2014 Jun 25;9(6):e100988. doi: 10.1371/journal.pone.0100988 (PMC4071031; doi:10.1371/journal.pone.0100988)
Supplement: Table S1 — Characteristics of autopsied patients. (DOCX) [file pone.0100988.s005.docx]

Supplementary Table S1 Characteristics of autopsied patients

| Group | Case No. | Age | Sex | Primary cause of death | HbA1c (%) (mmol/mol) | | Islet cell count (/cm^2^) | | |
| --- | --- | --- | --- | --- | --- | --- | --- | --- | --- |
|  |  |  |  |  |  |  | alpha | beta | total |
| Control | 1 | 81 | M | Lung cancer | 5.7 | (39) | 4181 | 6938 | 16710 |
|  | 2 | 80 | M | Bronchial pneumonia | NA |  | 1322 | 2476 | 6294 |
|  | 3 | 69 | M | Lung cancer | NA |  | 3153 | 5154 | 13290 |
|  | 4 | 65 | M | Gastric cancer | NA |  | 1591 | 2973 | 8776 |
|  | 5 | 48 | F | Gastric cancer | NA |  | 3500 | 2960 | 9611 |
|  | 6 | 60 | M | Esophageal cancer | NA |  | 4300 | 10393 | 33600 |
|  | 7 | 86 | M | Acute myocardial infarction | 6.2 | (44) | 3034 | 4872 | 15699 |
|  | 8 | 70 | F | Acute arterial occlusive disease | 5.4 | (36) | 7528 | 7441 | 16568 |
|  |  |  |  |  |  |  |  |  |  |
| T2DM | 1 | 67 | M | Bronchial pneumonia | 7.1 | (54) | 1247 | 1635 | 9040 |
|  | 2 | 78 | F | Hepatocellular carcinoma | 7.3 | (56) | 2279 | 2085 | 5424 |
|  | 3 | 70 | M | Panperitonitis | 6.7 | (49) | 2159 | 3370 | 10204 |
|  | 4 | 78 | M | Hepatocellular carcinoma | 6.3 | (45) | 1589 | 1630 | 4820 |
|  | 5 | 64 | F | Acute myelocytic leukemia | 8.8 | (73) | 2219 | 2798 | 9596 |
|  | 6 | 78 | M | Bronchial pneumonia | NA |  | 4175 | 5423 | 11622 |
|  | 7 | 52 | F | Ischemic colitis | NA |  | 537 | 968 | 2322 |
|  | 8 | 82 | F | Mitral valve stenosis | 8.0 | (64) | 3207 | 3385 | 12173 |
|  | 9 | 78 | M | Hemorrhagic gastric ulcer | 6.4 | (46) | 895 | 785 | 2666 |
|  | 10 | 64 | M | Bronchial pneumonia | 6.8 | (51) | 1808 | 2724 | 12522 |
|  | 11 | 77 | M | Sepsis | 7.6 | (60) | 399 | 603 | 2286 |
|  | 12 | 56 | M | Sepsis | 7.2 | (55) | 1093 | 1549 | 8172 |

| Group | Case No. |  | Duration of T2DM | Treatment (duration of medication use) | | |
| --- | --- | --- | --- | --- | --- | --- |
| T2DM | 1 |  | > 10 y | Insulin (> 3 m) |  |  |
|  | 2 |  | 10 y | Insulin (1 m), Hypoglycemics (NI) |  |  |
|  | 3 |  | 10 y | Insulin (7 d), Hypoglycemics (2 y 11 m) |  |  |
|  | 4 |  | 17 y | Insulin (NI) |  |  |
|  | 5 |  | 3 m | Insulin (1 m) |  |  |
|  | 6 |  | > 1 y 5 m | Insulin (> 1 y 5 m) |  |  |
|  | 7 |  | 22 y | Insulin (19 y) |  |  |
|  | 8 |  | 24 y | Insulin (> 1 y 2 m), Hypoglycemics (> 1 m) |  |  |
|  | 9 |  | NI | Insulin (1 m) |  |  |
|  | 10 |  | 4 y | Insulin (NI), Hypoglycemics (NI) |  |  |
|  | 11 |  | 4 y | Insulin (NI), Hypoglycemics (4 y) |  |  |
|  | 12 |  | > 8 y | Insulin (NI) |  |  |

NA, not available.

NI, not informative.

y, year; m, month.
